# Acute intra-cavity 4D flow cardiovascular magnetic resonance predicts long-term adverse remodelling following ST-elevation myocardial infarction

**DOI:** 10.1186/s12968-022-00889-7

**Published:** 2022-11-21

**Authors:** Arka Das, Christopher Kelly, Hadar Ben-Arzi, Rob J. van der Geest, Sven Plein, Erica Dall’Armellina

**Affiliations:** 1 Biomedical Imaging Science Department, Leeds Institute of Cardiovascular and Metabolic Medicine (LICAMM), University of Leeds, and Leeds Teaching Hospitals NHS Trust, Leeds, LS2 9JT UK; 2 Leiden University Medical Center, Leiden, The Netherlands

**Keywords:** 4D flow, Myocardial infarction, Adverse remodelling, In-plane kinetic energy

## Abstract

**Background:**

Despite advancements in percutaneous coronary intervention, a significant proportion of ST-elevation myocardial infarction (STEMI) survivors develop long-term adverse left ventricular (LV) remodelling, which is associated with poor prognosis. Adverse remodelling is difficult to predict, however four-dimensional (4D) flow cardiovascular magnetic resonance (CMR) can measure various aspects of LV intra-cavity flow beyond LV ejection fraction and is well equipped for exploring the underlying mechanical processes driving remodelling. The aim for this study was to compare acute 4D flow CMR parameters between patients who develop adverse remodelling with patients who do not.

**Methods:**

Fifty prospective ‘first-event’ STEMI patients underwent CMR 5 days post-reperfusion, which included cine-imaging, and 4D flow for assessing in-plane kinetic energy (KE), residual volume, peak-E and peak-A wave KE (indexed for LV end-diastolic volume [LVEDV]). All subjects underwent follow-up cine CMR imaging at 12 months to identify adverse remodelling (defined as 20% increase in LVEDV from baseline). Quantitative variables were compared using unpaired student’s t-test. Tests were deemed statistically significant when p < 0.05.

**Results:**

Patients who developed adverse LV remodelling by 12 months had significantly higher in-plane KE (54 ± 12 vs 42 ± 10%, p = 0.02), decreased proportion of direct flow (27 ± 9% vs 11 ± 4%, p < 0.01), increased proportion of delayed ejection flow (22 ± 9% vs 12 ± 2, p < 0.01) and increased proportion of residual volume after 2 consecutive cardiac cycles (64 ± 14 vs 34 ± 14%, p < 0.01), in their acute scan.

**Conclusion:**

Following STEMI, increased in-plane KE, reduced direct flow and increased residual volume in the acute scan were all associated with adverse LV remodelling at 12 months. Our results highlight the clinical utility of acute 4D flow in prognostic stratification in patients following myocardial infarction.

## Background

The sudden loss of contractility in the left ventricle (LV) following myocardial infarction (MI) results in an increased pre-load, which triggers various adaptive neurohormonal responses. Failure to normalise the increased pressures can result in progressive LV dilatation at the expense of left ventricular ejection fraction (LVEF). This process is known as adverse remodelling and is associated with poor prognosis [1]. The exact mechanisms driving the maladaptive changes are not fully understood, however sudden changes in intra-cavity blood flow are thought to play a substantial role in the pathophysiology.

Four-dimensional flow (4D-flow) cardiovascular magnetic resonance (CMR) imaging provides quantification of intra-cavity LV flow kinetic energy (KE) in three dimensions at different time points in the cardiac cycle (parameters are described in Table 1). By measuring components of systolic function beyond LVEF, it is well suited for exploring some of the mechanisms driving adverse remodelling. ‘In-plane KE %’ measures the proportion of blood flowing across the LV plane as opposed to ‘through-plane’ (blood flowing from the apex up and out the outflow tract as it leaves the LV) [2]. Previous authors have demonstrated MI patients to have increased in-plane KE than controls, and attributed this to the asymmetrical contraction of the LV cavity post-MI [3]. It is also possible to quantify and compare the proportion of blood that directly flows in and out the LV cavity vs blood that is retained in the cavity, and previous studies have shown that increased ‘residual volume’ of blood is associated with LV thrombus formation [4]. The impact of increased in-plane KE and residual volume on long-term LV remodelling hasn’t been investigated yet and remains unknown. The objective of this study was to perform 4D-flow CMR on patients shortly following ST-elevation myocardial infarction (STEMI) and compare the KE parameters between patients who undergo adverse remodelling at 12 months with patients that do not.

**Table 1:** Descriptions of left ventricular (LV) kinetic energy (KE) flow parameters used in this study

Parameter	Description
KE Parameters	
Average KE	The average KE of LV flow for the complete cardiac cycle
Minimal KE	The minimal KE of the LV flow at any time point during the complete cardiac cycle
Systolic KE	The average KE of the LV flow during systole
Systolic In-plane KE %	The proportion of flow that moves ‘in-plane’ across the LV plane rather than ‘through-plane’ from the apex to the LV outflow tract
Diastolic KE	The average KE of the LV flow during diastole
Components of LV washout over 2 cardiac cycles (measured in %)	
Direct flow	Blood that enters the LV during diastole and leaves the LV during systole in the analysed heartbeat
Retained volume	﻿Blood that enters the LV during diastole but does not leave during systole in the analysed heartbeat
Delayed ejection flow	﻿Blood that starts and resides inside the LV during diastole and leaves during systole
Residual volume	﻿Blood that resides within the LV for at least two cardiac cycles

All kinetic energy (KE) parameters were normalised to left ventricular (LV) end-diastolic volume (LVEDV) (presented as KE_iEDV_) [3]

## Methods

### Patient population

Fifty prospectively recruited STEMI patients reperfused by primary percutaneous coronary intervention (PCI) within 12 h of symptoms onset, underwent serial CMR scans at 5 ± 2 days and 378 ± 23 days following their index presentation. Study inclusion criteria were (a) MI as defined by current international guidelines [5], (b) revascularisation via primary PCI within 12 h after onset of symptoms and (c) no contraindications to CMR. Exclusion criteria were (a) previous revascularisation procedure (coronary artery bypass grafts or PCI), (b) known cardiomyopathy, (c) severe valvular heart disease, (d) atrial fibrillation and (e) haemodynamic instability lasting longer than 24 h following PCI and contraindication. The study protocol was approved by the institutional research ethics committee and complied with the Declaration of Helsinki. (NIHR 33963, REC 17/YH/0062).

### CMR

The study protocol included a CMR scan within 3–7 days of index presentation (acute scan), a second scan at 12 months. CMR examinations were performed on a 3T CMR system (Achieva TX, Philips Healthcare, Best, The Netherlands) equipped with a 32-channel cardiac phased array receiver coil, MultiTransmit technology and high-performance gradients with Gmax = 80mT/m and slew rate = 100 mT/m/ms. Survey images were used to plan vertical long-axis, horizontal long-axis, 3-chamber (LV outflow tract) views and the LV volume contiguous short axis stack. Cine imaging used a balanced steady-state free precession (bSSFP) pulse sequence (echo time (TE)/repetition time (TR)/flip angle 1.3 ms/2.6 ms/40°, spatial resolution 1.6 × 2.0 × 10 mm, typical temporal resolution 25 ms, slice thickness 8 mm, and 30 phases per cardiac cycle). Modified Look-Locker inversion recovery (MOLLI) to determine the T1-inversion time. LGE imaging was done at 15-min from gadolinium-based contrast injection, using phase sensitive inversion recovery (PSIR) spoiled gradient echo (GE) sequence (SENSE factor 1.7, typical TE/TR of 3.0/6.1 ms, flip angle of 25°, slice thickness of 10 mm and with Look-Locker scout determined T1-inversion time).

### 4D flow acquisition

An unnavigated free-breathing 4D flow data acquisition was planned in the trans-axial plane while ensuring complete ventricle coverage. A 3D echo planar imaging (EPI)-based, fast field echo (FFE) sequence was used with retrospective cardiac gating; 30 phases were reconstructed across the cardiac cycle. Sequence parameters were as follows: acquired voxel size = 3 × 3 × 3 mm^3^, reconstructed voxel size = 2.23 × 2.23 × 3 mm^3^, field of view (FOV) = 400 × 300 mm^2^, TR = 8.1 ms, TE = 3.5 ms, flip angle = 10°, number of signal averages = 1, VENC = 150 cm/s, EPI factor = 5. 4D flow data reconstruction, error and quality check methods were done as from previously published literature [3].

### Image analysis

Cine and LGE data were analyzed using cvi^42^ software (Circle Cardiovascular Imaging Inc, Calgary, Canada). Cine-images were used to derive LV volumes and LVEF, while LGE images were used to derive infarct size and identify microvascular obstruction (MVO). On LGE images, the threshold used for identifying infarcted tissue was set to 5 standard deviations above remote myocardial tissue signal intensity. MVO was defined as dark zones within an area of LGE at 15 min. Adverse remodelling was defined as an increase in LV end-diastolic volume (LVEDV) indexed for body surface area (LVEDVI) > 20% at 12 months from baseline [6]. 4D flow data was analyzed using the research software tool MASS (Leiden University Medical Center, Leiden, The Netherlands). Cine short-axis segmentation was used to define the boundaries of the region for LV blood flow parameter estimation. Prior to these calculations, spatial misalignment between the cine short-axis stack and 4D flow CMR data were corrected by rigid registration as previously described [3]. 4D flow measurements are described in Table 1.

### Statistical analysis

Statistical analyses were performed in SPSS (version 21.0; Statistical Package for the Social Sciences, International Business Machines, Inc., Armonk, New York, USA). Normality was checked using the Shapiro–Wilk test. Continuous variables are reported as mean ± SD. Comparison between quantitative variables was performed by independent-sample parametric (unpaired Student’s t-test) or non-parametric (Mann–Whitney) statistical test as appropriate. For comparing results from initial and repeated measurements, paired t-tests and ANOVA with Bonferroni post-hoc comparisons were used. Pearson correlation analysis was used to calculate the correlation coefficient between LVEF, infarct size and 4D flow parameters. All tests were assumed to be statistically significant when p < 0.05.

## Results

### Demographic characteristics

**Fig. 1:**
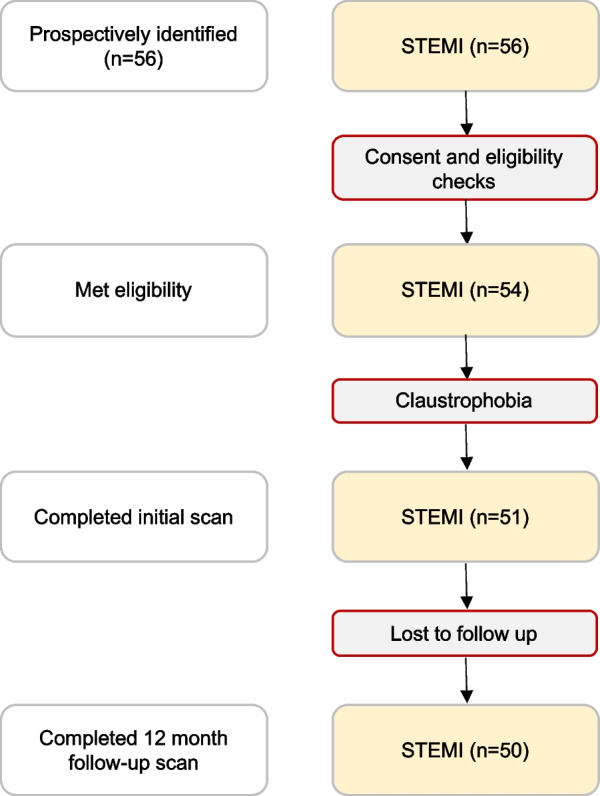
Flowchart of study enrolment. *STEMI*, ST elevation myocardial infarction

The study flowchart is shown in Fig. 1. Of the 54 patients prospectively recruited for the study, 3 patients experienced claustrophobia during the acute CMR scan, and 1 patient did not attend the follow-up CMR scan. The acute and 12-month CMR scans from the remaining 50 patients were used for statistical analysis. Patient demographics are displayed in Table 2. All patients received 12 months of dual antiplatelet and angiotensin converting enzyme inhibitor therapy, and all but 1 were on beta-blockers at the time of their 12-month scan. There was no significant difference in pre-scan systolic blood pressure between adverse and non-adverse remodellers (140 ± 34 vs 138 ± 28 mmHg, p = 0.52).

**Table 2:** Baseline characteristics of the study population

Patient Characteristics	All (n = 50)
Age (years)	57 ± 10
Sex	38:12 (M:F)
Risk Factors (No)	
Smoker	23
Hypertension	9
Diabetes	8
Family history	13
Peripheral vascular disease	2
Presenting characteristics	
Culprit coronary artery [No (%)]	
Left anterior descending	19 (38)
Left circumflex	10 (20)
Right coronary	21 (42)
Mean time from onset of symptoms to balloon (mins)	253 ± 189
Treatment [No (%)]	
Aspirin	50 (100)
Adenosine diphosphate receptor antagonist (Ticagrelor)	50 (100)
Angiotensin converting enzyme (ACE) inhibitor	50 (100)
Beta-blocker	49 (98)

### CMR measurements

The CMR characteristics are summarised in Table 3. In the acute scan, the mean LVEDV was 152 ± 41mls, with a mean LVEF was 43 ± 9% and mean infarct size of 14 ± 11 g. By 12 months, across the entire cohort, the mean LVEDV increased to 164 ± 52mls and 12 out of the 50 patients fulfilled the criteria for adverse remodelling.

**Table 3:** CMR and 4D Flow characteristics

CMR Characteristics	Acute scan (n = 50)	12-month scan (n = 50)	P value
Days from STEMI to scan	5 ± 2	378 ± 23	< 0.01
LVEDV (mls)	152 ± 41	164 ± 52	0.23
LVEDVI (mls/m^2^)	79 ± 18	85 ± 23	0.12
LVSV (mls)	64 ± 14	77 ± 15	< 0.01
LVEF (%)	43 ± 9	49 ± 10	< 0.01
Infarct size (g)	14 ± 11	9 ± 9	< 0.01
Infarct size (% of total LV mass)	22 ± 14	17 ± 17	< 0.01
MVO (n)	23 (46%)	-	-
MVO (g)	1.4 ± 2.8	0.0 ± 0.0	< 0.01
4D Flow CMR Characteristics from acute scan			
KE parameters (normalised for LVEDV)			
Full R-R average energy (μJ/ml)	9.4 ± 2.7	–	–
Full R-R minimal energy (μJ/ml)	1.3 ± 1.0	–	–
Systolic average energy (μJ/ml)	10.8 ± 3.9	–	–
Systolic In-plane flow (%)	45 ± 11	–	–
Diastolic average energy (μJ/ml)	8.7 ± 2.9	–	–
LV washout parameters		–	–
Direct flow (%)	42 ± 19	–	–
Retained volume (%)	16 ± 7	–	–
Delayed ejection flow (%)	19 ± 9	–	–
Residual volume (%)	23 ± 12	–	–

Values are displayed as mean ± standard deviation for continuous variables. *KE* kinetic energy, *LVEDV* left ventricular end diastolic volume, *LVEDVI* left ventricular end diastolic volume indexed for body surface area, *LVEF* left ventricular ejection fraction, *LVSV* left ventricular stroke volume. Infarct size was detected using late gadolinium enhancement

### Systolic LV flow measurements

On the acute scan, average systolic KE indexed for end-diastolic volume (KE_iEDV_) across the cohort was 10.8 ± 3.9 μJ/ml which is higher than previously reported values in acute MI patients by our group (9.2 ± 3.8 µJ/ml [3], but average systolic KE_iEDV_ was noted to decrease with worsening LV systolic function (ANOVA p = 0.01, Table 4).

**Table 4:** 4D flow CMR characteristics of patients with different degrees of left ventricular (LV) systolic impairment

4D Flow Parameters	Preserved LVEF (n = 11)	Mild (n = 24)	Moderate (n = 9)	Severe (n = 6)	ANOVA
KE parameters (normalised for LVEDV)					
LV R-R (μJ/ml)	11.1 ± 2.9	9.2 ± 2.8	8.7 ± 1.6	7.8 ± 1.8	0.06
Minimal (μJ/ml)	1.1 ± 0.4	1.4 ± 1.2	1.1 ± 0.6	1.5 ± 0.8	0.67
Average systolic (μJ/ml)	14.1 ± 3.4	10.9 ± 3.7	8.0 ± 2.4	8.3 ± 2.0	0.01
Systolic in-plane (%)	39 ± 8	42 ± 8	50 ± 12	60 ± 9	0.01
Average diastolic (μJ/ml)	9.1 ± 2.8	8.4 ± 2.8	9.6 ± 5.3	7.4 ± 2.0	0.46
LV washout parameters					
Direct flow (%)	35 ± 20	25 ± 10	18 ± 7	13 ± 10	0.860
Retained volume (%)	11 ± 6	17 ± 8	17 ± 6	11 ± 5	0.471
Delayed ejection flow (%)	24 ± 10	19 ± 7	18 ± 11	14 ± 5	0.003
Residual volume (%)	30 ± 26	39 ± 17	46 ± 15	62 ± 11	0.012

Values are displayed as mean ± standard deviation for continuous variables. *KE* kinetic energy, *EF* ejection fraction, *ANOVA* analysis of variance

**Fig. 2:**
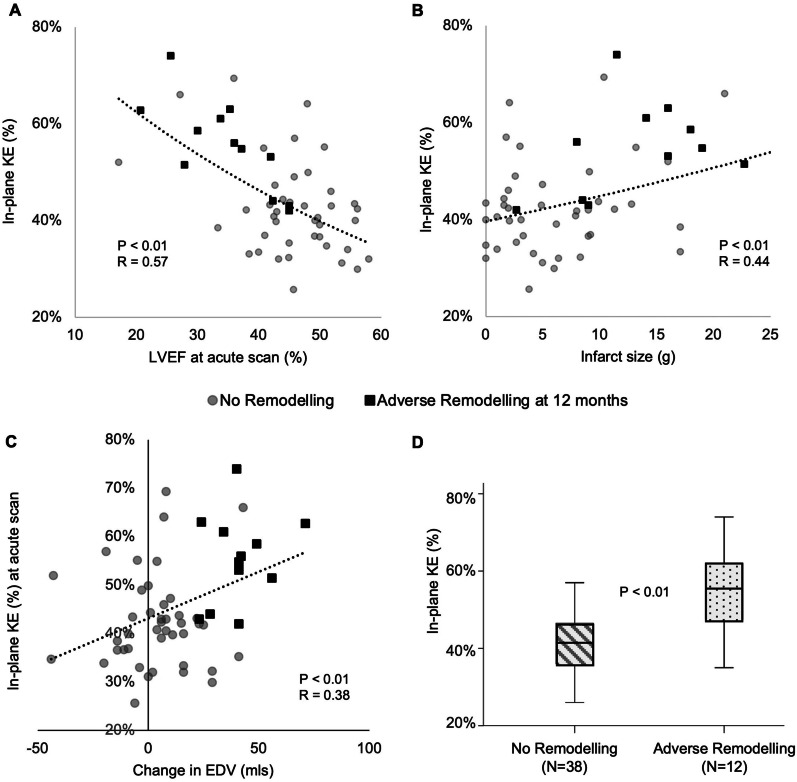
Associations between acute left ventricular (LV) systolic function, diastolic 4D flow parameters and LV remodelling. In the acute scan, lower LVEF correlated with lower peak E-wave **A** and higher peak A-wave **B** kinetic energy. Patients who went on to develop adverse remodelling at 12 months had significantly lower peak E-wave **C** and higher peak A-wave **D** kinetic energy during their acute scan. KE = kinetic energy; EDV = end-diastolic volume; LVEF = left ventricular ejection fraction

**Fig. 3:**
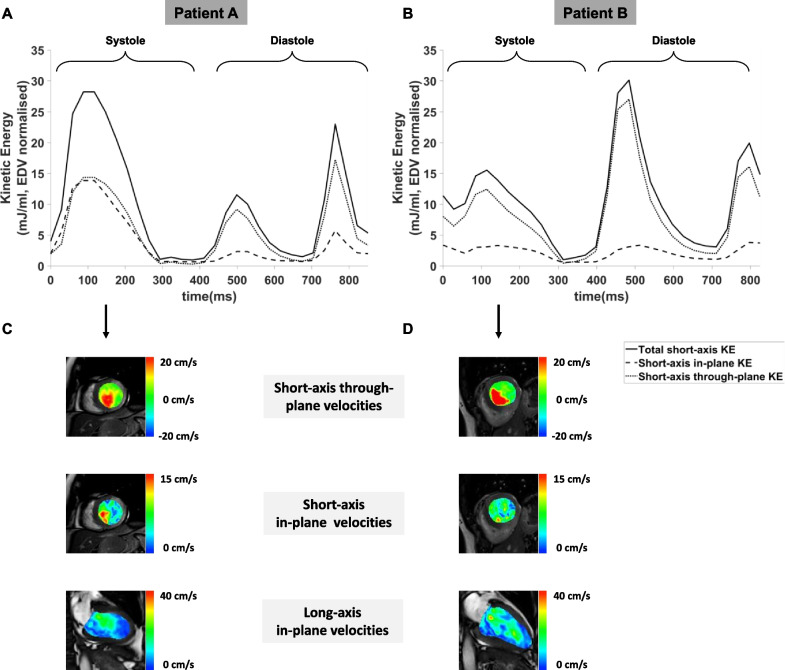
Representative time curves displaying In-plane vs through-plane kinetic energy in remodelled versus non-remodelled LV post-STEMI. **A** Case of a 46-year-old male with inferior ST-elevation myocardial infarction (STEMI), primary percutaneous coronary intervention (PCI) to right coronary artery (RCA) and acute left ventricular (LV) ejection fraction (LVEF) of 38%. 4D-flow CMR analysis show a proportion of systolic in-plane flow of 40%. By 12-months, this patient’s LV had undergone adverse remodelling (LVEF 31%). **B** Case of a 58-year- with inferior STEMI, PCI to RCA and acute LVEF of 46%. Compared to patient A, this patient had a lower proportion of in-plane flow during systole (21%). This patient did not undergo adverse remodelling by 12 months (LVEF 55%). Panel C and D shows velocity maps (short-axis through-plane, short-axis in-plane and long-axis in-plane) for the two patients respectively. Compared to patient B, patient A (who subsequently underwent adverse remodelling at 12 months) had lower through-plane, and higher in-plane velocities in the acute scan

Across the cohort, systolic in-plane KE % decreased with worse LV systolic function (ANOVA p < 0.01, Table 4 and Fig. 2a). Systolic in-plane KE also correlated with infarct size (p < 0.01, Fig. 2b). There was a significant correlation between in-plane KE in the acute scan and change in LVEDV over 12 months (p < 0.01, Fig. 2c). Patients who remodelled by 12 months (Table 5) had significantly higher systolic in-plane KE (54.0 ± 12.2% vs 42.5 ± 10.0%, p = 0.01 as shown on Fig. 2d), and lower average systolic KE (8.5 ± 3.1 μJ/ml vs 11.6 ± 3.7 μJ/ml, p = 0.01) on their acute scan than patients who did not undergo adverse remodelling. Figure 3 demonstrates time-curves of 2 separate subjects who both suffered an inferior STEMI with similar initial LVEF; patient A underwent adverse remodelling at 12 months while patient B does not. In comparison to patient B, on the acute scan, patient A has less through-plane and more in-plane KE during systole. There were no significant differences in acute diastolic in-plane KE between adverse and non-adverse remodellers (10 ± 5 vs 8 ± 4 μJ/ml, p = 0.22).

**Table 5:** Acute 4D flow CMR characteristics of patients with adverse remodeling at 12 months

Acute 4D Flow Parameters	No Remodelling at 12 months (n = 38)	Adverse Remodelling at 12 months (n = 12)	*P*-value
KE parameters (normalised for LVEDV)			
LV R-R (μJ/ml)	9.8 ± 2.7	8.1 ± 2.2	0.06
Minimal (μJ/ml)	1.3 ± 1.1	1.2 ± 0.7	0.56
Average Systolic (μJ/ml)	11.6 ± 3.7	8.5 ± 3.1	0.01
Systolic In-plane KE (%)	42.5 ± 10.0	54.0 ± 12.2	0.02
Average Diastolic (μJ/ml)	8.7 ± .2.7	8.7 ± 3.4	0.99
Components of intra-cavity flow (% of blood volume across 2 cardiac cycles)			
Direct flow	27 ± 9	11 ± 4	< 0.01
Retained volume	16 ± 6	14 ± 9	0.40
Delayed ejection flow	22 ± 9	12 ± 2	< 0.01
Residual volume	34 ± 12	64 ± 14	< 0.01

### LV wash-out parameters

**Fig. 4:**
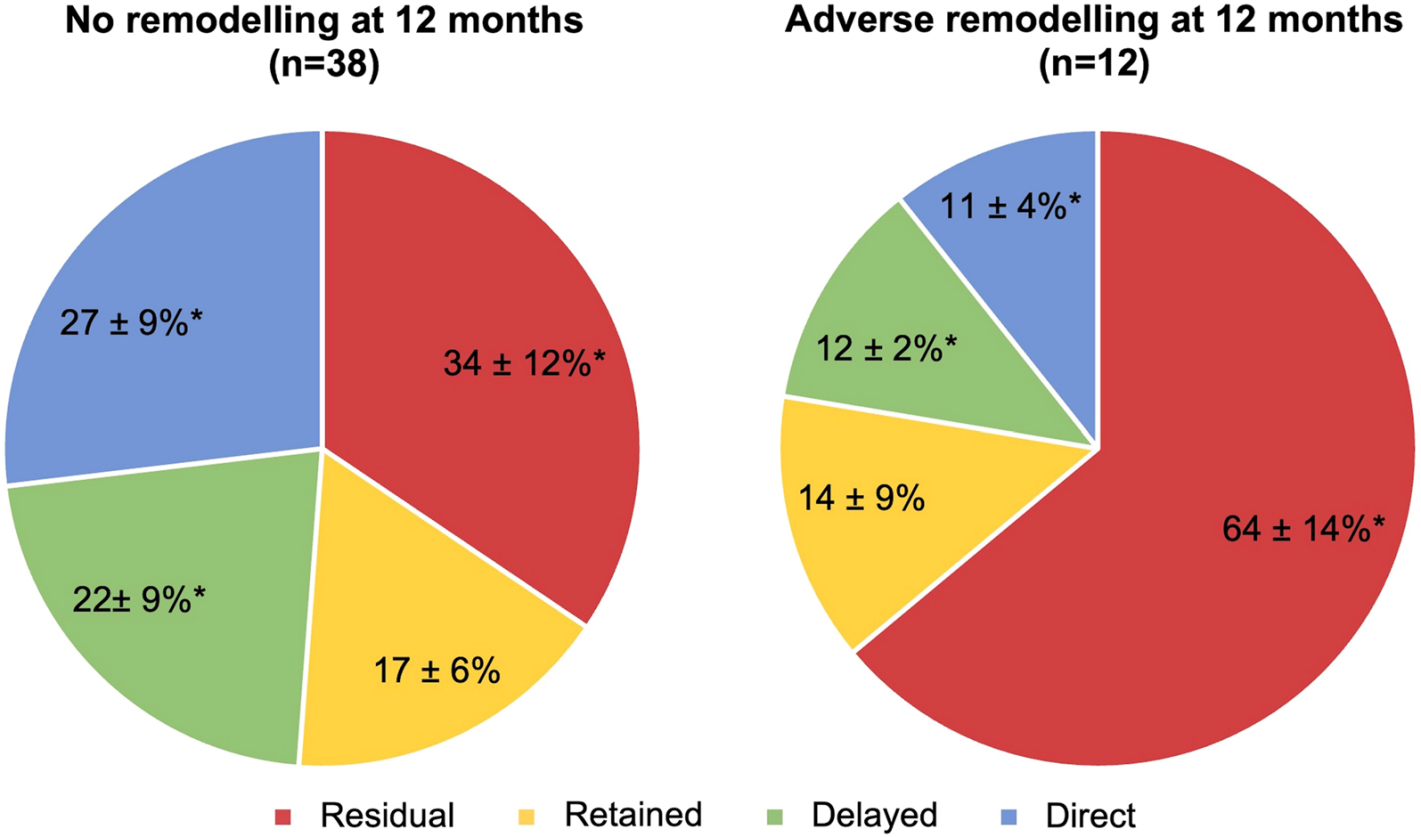
Components of intra-cavity flow (% of volume across 2 cardiac cycles). Patients who underwent adverse remodelling at 12-months had significantly reduced direct flow, increased delayed ejection flow and increased residual volume across 2 cardiac cycles in their acute 4D flow scan. * = significant difference between the 2 groups

When comparing the proportion of blood volume entering and leaving the LV cavity across 2 cardiac cycles, patients with more severe LV dysfunction tended to have less direct flow (p = 0.86), significantly higher proportion of delayed ejection flow (p = 0.003) and residual volume (p = 0.012), as shown in Table 4. Patients who had adverse remodelling at 12-months had significantly reduced direct flow (11 ± 4% vs 27 ± 9%, p < 0.01), increased delayed ejection flow (22 ± 9% vs 12 ± 2%, p < 0.01) and increased residual volume (64 ± 14% vs 34 ± 12%, p < 0.01) across 2 cardiac cycles in their acute 4D flow CMR scan, as shown in Table 5 and Fig. 4.

## Discussion

The complex mechanisms of adverse remodelling following MI, in particular the dynamic relationship between changes in cardiac anatomy and intraventricular flow have been only partly investigated to date and are not fully understood. To the best of our knowledge, this is the first study to demonstrate how acute changes in intra-cavity flow post-STEMI impacts on long-term adverse LV remodelling using CMR. The main findings include:

1. Acute In-plane KE % correlates with infarct size and LVEF and was significantly higher in patients who go on to develop adverse LV remodelling at 12 months.
2. Patients who adversely remodel had higher ‘residual volume’ across 2 cardiac cycles in their acute scan.

### In-plane KE

As contractility is lost in infarct segments, LV contraction becomes asymmetrical, meaning that wall tension is no longer homogeneously distributed in the cavity. Previous authors have attributed this to be the cause of greater ‘in-plane’ flow KE across the cavity [3]. This asymmetrical contraction is thought to exert ﻿heterogenous haemodynamic forces on the LV wall, which can cause stretching of the LV wall and lead to cavity dilatations over time [3, 4, 7, 8]. Garg et al. highlighted that larger infarct size correlated with greater in-plane KE following MI [3]. Results from our study matched this pattern, and in addition, demonstrates a direct association between in-plane KE and increase in LVEDV over 12-months, providing a link between infarct size, interventricular flow and long-term adverse remodelling. In-plane KE therefore provides an additional measurement of mechanistic function during systole beyond LVEF alone, which can be useful in predicting adverse remodelling. It is worth highlighting however that in-plane flow is likely one of several factors exerting pressure on the LV wall, and other contributing factors such as systemic blood pressure and LV end-diastolic pressures were not formally assessed in this study. In addition, distension of the LV wall during diastole is also likely to impact on cavity stretching and subsequent remodelling, however our results did not detect a significant difference in diastolic in-plane KE between adverse and non-adverse remodellers.

### Reduced LV wash-out

In a previous study, Stoll et al. performed 4D-flow CMR in heart failure patients (dilated cardiomyopathy and ischaemic heart disease) and demonstrated them to have decreased direct flow and increased residual volume following across 2 cardiac cycles than controls [2]. The degree of derangement in KE parameters correlated with myocardial dilatations and brain natriuretic peptide levels—neurohormones which are released in response to stretching of the cavity walls. Garg et al. also demonstrated how increased residual volume was predictive of the formation of LV thrombus, as a consequence of reduced diastolic LV wash-out [4]. Our results add to this finding by showing that decreased direct flow, and consequently increased residual volume was associated with long-term adverse remodelling. Like previous authors, we hypothesise that the increased stress placed on the LV cavity from the increased residual volume over time leads to stretching of the LV wall, with subsequent increase in cavity size [2, 4].

The impact of ischaemic injury on myocardial strain has been explored previously. Echocardiography based studies using echo-particle image velocimetry analysis and speckle-tracking, have shown that alterations in energy dissipation index and KE fluctuation index can be used to explain impairments in both LVEF and global wall motion indices following STEMI [9]. CMR strain imaging, which provides superior spatial resolution to speckle-tracking, have found that circumferential strain can be used to predict the recovery of long-term LV function, however associations between strain parameters and adverse LV remodelling remain unclear [10]. The impact of intraventricular flow and reduced LV-wash out on global and regional strain parameters has not yet been explored and may provide further mechanistic insights into the pathophysiology of adverse remodelling following MI.

### Limitations

Recruiting participants after STEMI for complex acute and longitudinal imaging was challenging, and the study sample size was therefore relatively small but aligned with similar studies [3]. The temporal resolution of the 4D flow CMR was 40 ms, which may affect the quality of KE and TD assessment. The LV geometry was defined by LV cine stack which was done using breath-hold technique while the 4D flow was done using free breathing. Hence, although spatial miss-registration was corrected for, other issues still remain including difference in heart rate and physiological conditions. This may have impact on the time-varying flow characteristics which could not be corrected for. Results from this study cannot be applied to patients with significant valvulopathy, cardiomyopathies and congenital heart disease.

## Conclusion

Acute 4D-flow imaging following STEMI allows for direct assessment of intra-cavity flow across the various stages of the cardiac cycle. Results from our study demonstrates increased in-plane KE, reduced direct flow and increased residual volume were all associated with adverse LV remodelling at 12 months. Our results highlight how 4D-flow CMR can complement currently available clinical and imaging biomarkers in prognostic risk stratification post-STEMI, prompting earlier initiation of aggressive heart failure therapy to those at highest risk of adverse outcomes.

## Data Availability

The data that support the findings of this study are available on request from the corresponding author, [ED].

## Abbreviations

4D: Four dimensional
bSSFP: Balanced steady-state free precession
CMR: Cardiovascular magnetic resonance
EPI: Echo planar imaging
FFE: Fast field echo
FOV: Field of view
GE: Gradient echo
KE: Kinetic energy
KE_iEDV_: Kenetic energy indexed to left ventricular end-diastolic volume
LGE: Late gadolinium enhancement
LV: Left ventricle/left ventricular
LVEDV: Left ventricular end-diastolic volume
LVEDVI: Left ventricular end-diastolic volume indexed for body surface area
LVEF: Left ventricular ejection fraction
LVSV: Left ventricular stroke volume
MI: Myocardial infarction
MOLLI: Modified Look-Locker inversion recovery
MVO: Microvascular obstruction
PCI: Percutaneous coronary intervention
PSIR: Phase sensitive inversion recovery
STEMI: ST-elevation myocardial infarction
TE: Echo time
TR: Repetition time
